# Self-esteem mediated relations between loneliness and social anxiety in Chinese adolescents with left-behind experience

**DOI:** 10.3389/fpsyg.2022.1014794

**Published:** 2022-11-08

**Authors:** Chen Chen, Liangyi Hu

**Affiliations:** Center for Educational Science and Technology, Beijing Normal University, Zhuhai, China

**Keywords:** loneliness, social anxiety, self-esteem, left-behind children, mediating effect

## Abstract

Although research examining loneliness and social anxiety has been conducted, few studies have explored pathways from loneliness at home to social anxiety at school in Chinese left-behind children. The study attempts to explore associations between loneliness at home and social anxiety at school and to examine roles of self-esteem in those relationships among a sample of Chinese left-behind children. Date were collected from 303 left-behind children, aged 10–14 years, and the Chinese versions of Children’s Loneliness Scale, Social Anxiety Scale, and Rosenberg Self-esteem Scale were used to measure loneliness at home, social anxiety at school, and self-esteem, respectively. Results showed that loneliness at home was positively associated with social anxiety at school; self-esteem played a partial mediation role in associations between loneliness at home and social anxiety at school. Findings suggest that high levels of self-esteem may influence pathways from loneliness at home to social anxiety at school in Chinese left-behind children, and increasing levels of self-esteem may be used in preventions for loneliness of Chinese left-behind children.

## Introduction

With the acceleration of urbanization in China, more and more young adults leave the land to find work in towns and cities which leads to some social problems. Typically, left-behind children whose mother or father or both parents leave the countryside to work in towns and cities for more than 6 months are a special group of children that needs much more attention (Fan et al., 2018). Millions of children have been left behind in countryside each year (Fan et al., 2017), and left-behind experiences is related to many aspects of child development in short-and long-terms (Qi and Yang, 2020; Shao and Zhang, 2020), such as, behavior problems (e.g., Yang and Huang, 2022), peer relationships (e.g., Xie et al., 2020), and attention bias (e.g., Yang and Chen, 2017).

In light of both the basic and applied value of a clearer account of how left-behind experiences is related to child development, studies which explore pathways from family life to school life of left-behind children need further exploration. Notably, extensive research has suggested that left-behind children felt much more loneliness (e.g., Ren et al., 2020) and social anxiety (Li M. et al., 2020) than children without left-behind experiences, and loneliness was associated with social anxiety (Alyson and Cheryl, 2015; Baytemirand and Yıldız, 2017; Chen et al., 2021). Moreover, a growing body of studies have indicated that self-esteem was related to loneliness (e.g., Yıldız and Karadaş, 2017) and social anxiety (e.g., Abdollahi and Talib, 2016). However, few studies have explored relationships between loneliness at home, social anxiety at school, and self-esteem in left-behind children.

The attachment theory posits that patterns of relationships between parent (s) and child may be generalized to other relationships or situations (Crittenden, 1985), which may provide a framework for exploring studies in left-behind children. Specifically, lack interactions between parents and left-behind children may contribute to high levels of loneliness at home (Zhao et al., 2021), which may influence the levels of self-esteem (Feng et al., 2019), and in turn, lead to high levels of social anxiety at school (Chen et al., 2021). Therefore, we attempt to explore pathways from loneliness at home to social anxiety at school and to examine roles of self-esteem in those pathways in Chinese left-behind children.

### The relationships between loneliness and social anxiety

Loneliness is an enduring condition of emotional distress, which influenced by lacking interactions and supports with/from parents or others (Zhang, 2011; Alkan, 2014; Stickley et al., 2015). Left-behind children may have less interactions with parents (Fan et al., 2016), and other family members (e.g., grandparents) may not provide psychological supports for children (Li et al., 2013), which may contribute to high levels of loneliness (Li J. et al., 2021). Social anxiety, as a developmental maladaptation, contains emotional and behavior problems, such as over anxious about communicating with strange others, which typically occurs in early-to mid-adolescence (Fehm et al., 2005). Left-behind children may suffer high levels of social anxiety during childhood (Li et al., 2019).

Meanwhile, associations between loneliness and social anxiety in left-behind children have been explored by a growing body of studies. For example, Duan (2014) found that loneliness was positively associated with social anxiety in 187 Chinese left-behind children. Wang and Yao (2020) confirmed these results in 442 Chinese left-behind children. Children with left-behind experiences have less interactions with parent (s), which may contribute to loneliness at home (Fan and Wu, 2020), and loneliness in family life may spread to school life, which may increase the levels of social anxiety at school (Yuan et al., 2014; Li and Ran, 2018). However, few studies have explored relationships between loneliness at home and social anxiety at school in left-behind children. Therefore, the current study attempts to explore relationships between loneliness at home and social anxiety at school in a sample of Chinese left-behind children. We hypothesize that loneliness at home is positively associated with social anxiety at school in a sample of Chinese left-behind children.

#### Self-esteem as a mediator

The relationships between loneliness and self-esteem have been explored by a growing body of studies. For example, Li L. et al. (2021) found that loneliness was negatively associated with self-esteem in a sample of Chinese children. Song et al. (2017) also found loneliness was negatively associated with self-esteem in a sample of Chinese left-behind children, and Chen and Yu (2022) confirmed these relationships in a sample of overseas Chinese left-behind children.

Moreover, some studies have found relationships between self-esteem and social anxiety. For example, Wu et al. (2020) found that self-esteem was negatively associated with social anxiety with a sample of Chinese high school students. You et al. (2019) confirmed these relationships based on a sample of Chinese college students. Meanwhile, Wang et al. (2008) reported self-esteem was negatively associated with social anxiety in Chinese left-behind children.

Although the associations between loneliness, self-esteem, and social anxiety have been explored by previous studies, few studies have explored roles of self-esteem in the relationships between loneliness and social anxiety, particularly in a sample of Chinese left-behind children. Left-behind children may have less interactions with parents that may contribute to high levels of loneliness at home, which may contribute to low levels of self-esteem, and these low levels of self-esteem may spill over to school life which contribute to high levels of social anxiety at school. We hypothesize, thus, that self-esteem mediates the relationships between loneliness at home and social anxiety at school in Chinese left-behind children.

#### The current study

Although the associations among loneliness, social anxiety, and self-esteem have been explored by extensive studies, pathways from loneliness at home to social anxiety at school in Chinese left-behind children need further exploration. Moreover, left-behind children is an important issue for social development in China, which may raise the importance for exploring Chinese left-behind children. Therefore, the current study attempts to explore relationships between loneliness at home and social anxiety at school and to examine roles of self-esteem in those relationships in a sample of Chinese left-behind children, which may confirm the spillover effects of family life. We hypothesize that loneliness at home is positively associated with social anxiety at school, and self-esteem mediates relationships between loneliness at home and social anxiety at school among Chinese left-behind children.

## Materials and methods

### Participants

A total of 303 left-behind children whose father or mother or both parents were not live with them for over 6 months were recruited from two primary schools in Jiangsu Province, China, with a convenience sampling. Of those participants, 49.8% of them were girls (*n* = 151). The mean age was 11.68 years (SD = 0.83), with a range of 10–14 years. The detailed demographic information of participants is presented in Table 1.

**Table 1:** Demographic information of participants (N = 303).

		Adolescents with left-behind experience	
		N	%
Gender	Male	152	50.2
	Female	151	49.8
Child number	1	95	31.4
	>1	208	68.6
Left-behind type	Mother at home	145	47.9
	Father at home	19	6.3
	Live with grandparents without parents	139	3.3
Family monthly income	Less than 1,000	18	5.9
	1,000 to 3,000	94	31.0
	3,001 to 5,000	115	38.0
	More than 5,000	76	25.1
Father’s education year	Less than 6 years	53	17.5
	6 to 9 years	131	43.2
	9 to 12 years	87	28.7
	More than 12 years	32	10.6
Mother’s education year	Less than 6 years	80	26.4
	6 to 9 years	125	41.3
	9 to 12 years	68	22.4
	More than 12 years	30	9.9

Mother at home: children live with mothers only at countryside; father at home: children live with fathers only at countryside; live with grandparents without parents: children live with grandparents without mothers or fathers at countryside.

### Measures

Children’s Loneliness Scale (CLS). The CLS, developed by Asher et al. (1984), is a 16-item self-reported scale used to access loneliness for children (e.g., *It is hard for me to make friends*). The way of scoring each response is a 5-point scale (from 1 = *never* to 5 = *always*). Individuals who get high score of this scale may have high levels of loneliness. The Chinese version of CLS was administered in the current study to access loneliness at home (Wang et al., 1999), and the Cronbach’s Alpha of this scale was.82 in the present study. According to the item parching (Bian et al., 2007) which is used to make better fit for the models, the scale of loneliness was to broken into 4 parts based on factor analysis, loneliness 1 (LO1), loneliness 2 (LO2), loneliness 3 (LO3) and loneliness 4 (LO4).

Social Anxiety Scale for Children (SASC; La Greca et al., 1988). The SASC is a 10-item self-reported tool to access social anxiety for children, including subscales of fear of negative evaluation (FNE; e.g., *I am afraid of been teased*) and social avoidance and distress (SAD; e.g., *I feel nervous when taking to strange children*). The way of scoring each response is a 3-point scale (from 0 = *never* to 2 = *always*). Individuals who get higher scores may suffer higher levels of social anxiety. The Chinese version of SASC was administered in the current study to access social anxiety at school (Ma, 1993), and the Cronbach Alpha of this scale was.73 in the present study.

Rosenberg Self-Esteem Scale (SES). The SES, developed by Rosenberg (1965), is a 10-item self-reported questionnaire used to access self-esteem in this study (e.g., *I have lots of good qualities*). Participants were asked to rate each item on a 4-point scale (from 1 = *very conformity* to 4 = *very inconformity*). Ratings were averaged to form a total score on this measure and individuals who get higher scores have higher self-esteem (SE). The Chinese adaption of the SES was administered in the current study (Ji and Yu, 1999), and the Cronbach’s Alpha of this scale was.73 in the present study.

Covariate variables. The demographic variables of participants were controlled using regression analysis during the data analysis, such as gender, grade, the number of children in the family, left-behind type, parents’ education years, and family income.

### Procedure

There were several steps to conduct the current study. First, the first author chose two primary schools in Jiangsu Province with a convenience sampling, and then presented aims and processes of the current study to the headmasters, and received their permission to conduct the current study in their schools. Second, the first author randomly chose two classes for fourth, fifth, and sixth grade, respectively, in each primary school, and totally 12 classes were recruited in the current study. Third, a total of 303 left-behind children finished the questionnaires within 20 min, and all of left-behind children, caregivers, and teachers signed informed consent before data collecting process. All participants have a right to decline participation without any negative influences. Finally, participants received a small gift worth 5 RMB ($0.75). The study was also approved by the ethics committee of the authors’ institution (Code: 202102230004) and materials and process in the current study were safe for participants.

### Data analysis

Before data analysis, normality, missing values, and outliers were examined and the questionnaires with missing data (15%) were excluded (Davey and Savla, 2010). Pearson correlation analysis and independent *t*-test were done to examine relations among all study variables. All tests were two-tailed for significance, and significance (*p* value) was set at.05. Structural equation modeling (SEM) and Bootstrapping Method were performed to examine relationships and mediating models among all variables. The goodness of model fit was accessed using chi-square statistics (*χ^2^/df*), Root Mean Square Error of Approximation (RMSEA), and Standardized Root-Mean-Square Residual (SRMR). Fit indexes were the Comparative Fit Index (CFI), Goodness of Fit Index (GFI), the Relative Fit Index (RFI), Normed Fit Index (NFI), and Tucker Lewis Index (TLI). A *χ^2^/df* (degrees of freedom) less than 3 is considered a good fit; less than or equal to 5 indicates an acceptable fit. Moreover, CFI, TLI, GFI, NFI, and RFI values of.90 or higher indicate a good fit. RMSEA and SRMR values of less than 0.08 are considered a close fit and values less than.05 indicate a good fit (Kline, 2011).

## Results

### Descriptive analysis

Descriptive statistics and correlations are presented in Table 2. Loneliness at home was positively correlated with social anxiety at school (*r* = 0.47, *p* < 0.01); self-esteem was negatively correlated with loneliness at home and social anxiety at school (*r* = −0.43, and *r* = −0.34, *p* < 0.01). Additionally, the common method variance did not influence the result in the current study (17.51% < 40%).

**Table 2:** Means, standard deviations, and correlations between study variables (N = 303).

Variables	M SD		1	2	3
1. Loneliness	2.04	0.54			1
2. Social anxiety	1.54	0.36	0.47^**^		
3. Self-esteem	2.89	0.42	−0.43^**^	−0.34^**^	

**p < 0.01.

### The relationships between loneliness at home and social anxiety at school

Average scores were used to determine structures of loneliness at home, social anxiety at school, and self-esteem, and all coefficients were standardized estimates. SEM was used to make a direct model which to measure relationships between loneliness at home and social anxiety at school. The results indicated that loneliness at home was positively associated with social anxiety at school in left-behind children (*β* = 0.65, SE = 0.07, C.R. = 7.13, *p* < 0.001). All model fit indexes were acceptably (see Table 3).

**Table 3:** Model fit indexes.

	*χ^2^*/*df*	SRMR	RMSEA	GFI	NFI	RFI	IFI	TLI	CFI
Direct model	2.93	0.02	0.06	0.97	0.96	0.92	0.97	0.94	0.97
Mediation model	2.40	0.01	0.05	0.97	0.95	0.91	0.97	0.95	0.97

### Mediating effect of self-esteem in relationships between loneliness at home and social anxiety at school

**Figure 1:**
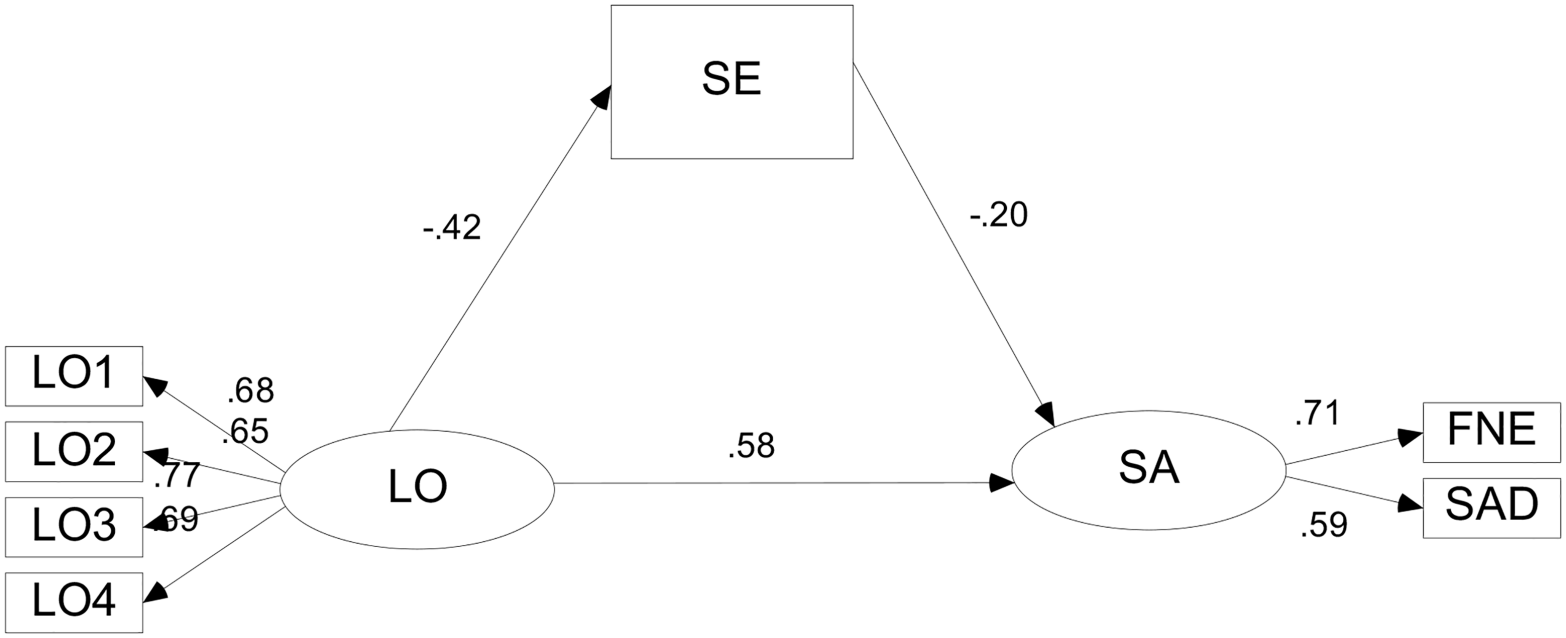
Standardized parameter estimates of the structural model demonstrating effects of loneliness on social anxiety *via* self-esteem. LO: Loneliness at home; SA: Social anxiety at school; SE: Self-esteem; *n* = 303.

The mediation analysis has two steps according to Wen and his colleagues (Wen et al., 2005). One step is to develop a direct model; the second step is to develop a mediation model which input the mediator into the direct model. As we had noted above, the direct model between loneliness at home and social anxiety at school in left-behind children was verified. Then the mediation model was developed with Bootstrapping Method Bias-corrected percentile method. The SEM results showed that structural models provided acceptable good fit to the data (see Table 3) and the SEM results indicating relationships between variables are presented in Figure 1.

In the mediation model, loneliness at home was positively associated with social anxiety at school (*β* = 0.58, *p* < 0.001) and negatively associated with self-esteem (*β* = − 0.42, *p* < 0.001). Self-esteem was negatively associated with social anxiety at school (*β* = − 0.20, *p* < 0.001). The results of Bias-corrected percentile method showed that the 95% confidence intervals (CI) of indirect effects was [0.02, 0.10], and the 95% CI of direct effects was [0.31, 0.49]. Self-esteem partial mediated relationships between loneliness at home and social anxiety at school among left-behind children.

## Discussion

The current study explored relationships between loneliness at home and social anxiety at school and examined roles of self-esteem in those relationships in a sample of Chinese left-behind children, which may broaden studies of left-behind children and provide suggestions for protecting left-behind children. The results showed that loneliness at home was positively associated with social anxiety at school and self-esteem partial mediated those relationships in Chinese left-behind children.

The results showed that loneliness at home was positively associated with social anxiety at school in Chinese left-behind children, which were consistent with previous studies (Yuan et al., 2014). Left-behind children who have less interactions between parents may have high levels of loneliness at home and less communication skills (Zhan and Chen, 2013), which may estrange themselves from others at school, in turn, may increase the levels of social anxiety in school lives (Li and Ran, 2018). Meanwhile, according to attachment theory, left-behind children who have less interactions with parents may have biased attachment patterns (e.g., unsecure attachment; Zou et al., 2022), which may influence internal working models about self and others and resource control strategies (Chen and Chang, 2012), in turn, contribute to high levels of social anxiety at school. These findings suggest that experiences in family, such as left-behind experiences, may influence individuals’ school life (e.g., social anxiety; Chang and Lu, 2018).

Moreover, the results showed that self-esteem partial mediated relationships between loneliness at home and social anxiety at school in Chinese left-behind children, which were in lined with our hypothesis. Less interactions with parents may contribute to high levels of loneliness in left-behind children and left-behind children with high levels of loneliness may feel high levels of personal discrimination (Miao et al., 2015), which may contribute to low levels of self-esteem (Li L. et al., 2020), and these levels of self-esteem may increase the levels of social anxiety at school life. Further, interactions with parents may influence development of self-esteem, which suggests that left-behind experiences may impair self-esteem, and the impaired self-esteem may contribute to high levels of social anxiety at school. These findings suggest that loneliness not only has direct effects on social anxiety, but also has indirect effects on it *via* self-esteem in Chinese left-behind children.

The implications of these findings are significant, underscoring the role self-esteem involved in adjustment of left-behind children and pointing to potentially modifiable mechanisms. Specifically, increasing the levels of self-esteem may reduce levels of social anxiety at school in left-behind children. For example, teachers may give more supports for left-behind children, which may raise their skills for interpersonal communication. Moreover, strengthening relationships within peers may increase self-esteem of children with left-behind experiences (Xie et al., 2020). Additionally, we should pay attention to left-behind children, and call for parents giving children the high quality of companion.

Several limitations should be acknowledged in this study. First, self-reported scales were only used to measure all variables in the present study, which may contribute to the biased information from the participants; several approaches should be applied to collect data in future research to ensure the accuracy of the information, such as experiments, questionnaires or interviews. Second, cross-sectional design was used to examine relationships between loneliness at home and social anxiety at school, which may not verify the relations causally. Longitudinal studies should be conducted to confirm the relationships between loneliness at home, social anxiety at school, and self-esteem. Third, the current study did not compare outcomes of children based on kinds of left-behind (e.g., mother or father at home), which may ignore the differences in outcomes based on kinds of left-behind. Future studies should take left-behind types into consideration and provide efficient strategies for protecting left-behind children.

## Conclusion

The current study explored relationships between loneliness at home and social anxiety at school and examined roles of self-esteem in those relationships in a sample of Chinese left-behind children. The results showed that loneliness at home was positively associated with social anxiety at school and self-esteem partial mediated those relationships in Chinese left-behind children. Findings suggest that loneliness not only has direct effects on social anxiety, but also has indirect effects on it *via* self-esteem in Chinese left-behind children. For example, the government and schools may use strategies to increase levels of self-esteem in left-behind children, which may decrease levels of social anxiety at school. Moreover, the current study may also broaden the studies of Chinese left-behind children that combination of family and school life, which may provide relative new perspectives for the field of study.

## Data availability statement

The original contributions presented in the study are included in the article/Supplementary materials; further inquiries can be directed to the corresponding author.

## Ethics statement

The studies involving human participants were reviewed and approved by Beijing Normal University. Written informed consent to participate in this study was provided by the participants’ legal guardian/next of kin.

## Author contributions

CC designed the study, wrote and revised the introduction and discussion of the manuscript, and completed the data analysis. LH wrote and revised the method and results of the manuscript. All authors contributed to the article and approved the submitted version.

## Funding

This study was supported by Ministry of Education of the People’s Republic of China under Grant [number 21YJC880005], Department of Education of Guangdong province [2021GXJK199], and Beijing Normal University at Zhuhai (28817–111032102).

## Conflict of interest

The authors declare that the research was conducted in the absence of any commercial or financial relationships that could be construed as a potential conflict of interest.

## Publisher’s note

All claims expressed in this article are solely those of the authors and do not necessarily represent those of their affiliated organizations, or those of the publisher, the editors and the reviewers. Any product that may be evaluated in this article, or claim that may be made by its manufacturer, is not guaranteed or endorsed by the publisher.
